# Supplementary material for: The role of mental health in the relationship between nursing care satisfaction with nurse-patient relational care in Chinese emergency department nursing
Source: PLoS One. 2024 Sep 3;19(9):e0309800. doi: 10.1371/journal.pone.0309800 (PMC11371248; doi:10.1371/journal.pone.0309800)
Supplement: Attachment — Submitted filename: Response to Reviewers.docx [file pone.0309800.s001.docx]

I thank you, dear reviewers. Your valuable comments can make me learn more and more. I tried my best to do the revisions according to your comments.

Also, we tried to edit the entire text.

Reviewer comment: Keywords："mental health" should read "Mental health".

we corrected (line 41).

Reviewer comment: Line 28 - 38, page 2: Pay attention to the norms of punctuation, with spaces before and after "=" and "<".

According to one of the articles that was recently published in PLOS ONE journal (Waddington EE, Allison DJ, Calabrese EM, Pekos C, Lee A, et al. (2024) Orienteering combines vigorous-intensity exercise with navigation to improve human cognition and increase brain-derived neurotrophic factor. PLOS ONE 19(5): e0303785. <https://doi.org/10.1371/journal.pone.0303785>), I edited the punctuation in the entire text.

Reviewer comment:  Overall: The abstract requires a brief overview of the background, purpose, methods, results, and conclusions of the article. What's your conclusion?

we added conclusion and edited.

Reviewer comment:  Line 61 - 63, page 4: “hospitals and many healthcare centers strive to fulfill patients' needs through the provision of quality services” Is it possible to give some relevant examples so that readers can understand.

We added (lines 46-50).

Reviewer comment:   Line 76 - 77, page 4: “Emergency departments have a distinct advantage in influencing a patient's initial perception as they navigate through the healthcare system” How is the emergency department different from other departments and can it be explained?

A visit to the emergency department is usually the first time a patient interacts with a hospital system. Thus, this is a special opportunity to make a positive first impression.

We added these sentences (lines 65-67).

Reviewer comment: Line 98 - 103, page 5: Are all tertiary hospitals in the area included?

Yes, all Level-III hospitals were included in the study. All the cities of Hubei province except for three cities had this model of hospitals.

Reviewer comment: Line 99, page 5: “November 10 to December 30, 2023.” When were the surveys of nurses and patients respectively conducted? Please be specific.

We added (lines 112 to 115).

Reviewer comment:  Line 102, page 5: “only Grade III hospitals were selected” Why choose more than just secondary hospitals? Why choose only tertiary hospitals? What are the advantages of tertiary hospitals over other levels of hospitals for this study?

We had to choose a hospital model to match the situation. Therefore, this question arose in each of the hospitals we chose. Therefore, choosing these types of hospitals in our research does not have a special advantage. In the future, research can be done according to the quality level of hospitals and comparison of services.

Reviewer comment: Line 104, page 5: Why were emergency department nurses used as survey respondents? And not other unit nurses? How is the emergency department different from other departments?

A visit to the emergency department is usually the first time a patient interacts with a hospital system. Thus, this is a special opportunity to make a positive first impression. Therefore, the nurses who work in these departments are among the first medical staff of the hospital who deal with the patient.

We added these sentences (lines 65-67).

Reviewer comment: Line 104 - 108, page 5: “with at least one year of working experienc”“nurses who had taken a leave for more than six months in the previous year due to different reasons” Why the one-year and six-month timeframes? Is there a basis for this? If so, please explain.

This time period was chosen according to a pattern taken from other articles.

Reviewer comment:  Line 129 - 130, page 7 - 8: 1) Pay attention to the norms of punctuation, please verify that punctuation is used correctly throughout the text. 2) Please use a 3-wire meter. 3) Personally, I don't think there is a great need to set up a table to account for this information, and it's fine to account for it directly in the body of the text.

Yes, according to your comment, I removed the table and entered the content in the text (lines 116-118).

Line 131 - 135, page 8: Is a demographic table available? If not, it is recommended that a demographic table be drawn up so that the demographic characteristics of nurses and patients are displayed more clearly:

I added Table 1.

Line 138 - 139, page 8: The result of the application? Good or bad? Please elaborate.

This questionnaire is not classified. In addition, we were looking for quantitative data to evaluate its relationship with mental health.

Line 143, page 8: “Cronbach's α coefficient” Note that the full name is alpha.:

I corrected (lines 133, 141, 152).

Line 164 - 169, page 9: 1) chi-square test requires italics. Please correct.

I corrected.

Line 170 - 172, page 10: What is the recommended range to which these values apply? What is the literature on which they are based? Please provide details.

I added lines 162 to 164.

Line 174, page 10: Are 532 nurses and 532 patients the final sample size? Were there any missing samples during the survey? How were the missing samples handled?

I added lines 166 to 170.

Pay attention to the norms of punctuation, please verify that punctuation is used correctly throughout the text.

We edited it.
